# Comparison of monensin sodium sources for finishing beef cattle

**DOI:** 10.1093/tas/txab090

**Published:** 2021-05-16

**Authors:** Taylor C Husz, Wyatt N Smith, Caleb G Lockard, Megan N Homolka, Peter T Anderson, Wes W Gentry, Joel D Sugg, Kenneth D Casey, Jenny S Jennings

**Affiliations:** 1 Texas A&M AgriLife Research, Amarillo, TX 79106, USA; 2 Midwest PMS, LLC, Firestone, CO 80504, USA

**Keywords:** methane, monensin sodium, volatile fatty acids

## Abstract

The objective of this study was to evaluate the ruminal fermentation characteristics of ruminally fistulated beef steers consuming a steam-flaked corn (**SFC**) or dry-rolled corn (**DRC**) based diet containing either Rumensin 90 (**RUM**; Elanco, Greenfield, IN), or Monovet 90 (**MV**; Huvepharma, Peachtree City, GA). Six ruminally fistulated steers (657.7 kg ± 72.6) housed individually were used in a 6 × 6 Latin square design with 2 × 3 factorial treatment arrangement. Each of the 6 periods were 15 d with 14 d for diet adaptation and 1 d of rumen fluid collections. Dietary treatments were DRC without monensin sodium (**DRC-C**), SFC without monensin sodium (**SFC-C**), DRC with Rumensin 90 (**DRC-R**), DRC with Monovet 90 (**DRC-MV**), SFC with Rumensin 90 (**SFC-R**), and SFC with Monovet 90 (**SFC-MV**). Rumen contents and fluid were collected through the fistula of each animal at 0, 3, 6, 12, and 24 h on d 15 of each period. Rumen fluid collected at 6 h post-feeding each period was used for in vitro analyses. Steer was the experimental unit and the model included fixed effects of grain processing, additive, and grain processing × additive. Total gas produced was composited from each in vitro bottle into a gas collection bag for the 48-h determination of methane concentration. No differences were detected for DMI (*P* = 0.81). Ruminal pH did not differ for the control or additive treatments (*P* = 0.33). However, ruminal pH was lower (*P* < 0.01) with SFC compared to DRC. There was a significant difference in acetate to propionate ratio for grain type (*P *= 0.01) and a tendency for additive inclusion (*P *= 0.06). Additive inclusion reduced methane proportion of total gas compared to control treatments (*P* ≤ 0.01). Overall, monensin sodium reduced methane concentration though source had no effect on DMI or ruminal pH.

## INTRODUCTION

Monensin sodium is a widely used ionophore as it has associated benefits to improve feed efficiency and nitrogen (**N**) metabolism in beef cattle (Bergen and Bates, 1984; Schelling, 1984). These positive effects on energetic efficiency are the results of increased ruminal propionate production (Bergen and Bates, 1984) and limiting the growth of Gram-positive bacteria (McGuffey et al., 2001; Wood et al., 2016). Monensin may also help mitigate ruminal acidosis as it can inhibit lactic acid-producing bacteria in the rumen (Dennis et al., 1981; Wood et al., 2016), stabilize dry matter intake (**DMI**; Duffield et al., 2012; Wood et al., 2016), and increase gain to feed (**G:F**; Ellis et al., 2012; Wood et al., 2016). Monensin sodium has also been reported to decrease ruminal frothy bloat by depressing rumen fluid viscosity and decreased froth from protozoa and Gram-positive *S. bovis* (Bergen and Bates, 1984; Schelling, 1984).

An increase in propionate production due to monensin sodium will result in a decrease in available substrate for methanogens (Thornton and Owens, 1981; Ellis et al., 2012; Melchior et al., 2018). This decrease in methanogens will overall reduce the amount of energy lost as methane (**CH**_**4**_; Thornton and Owens, 1981; Ellis et al., 2012). Currently, there are two monensin sodium sources commercially available to the beef industry in the U.S., Rumensin 90 (**RUM**; Elanco, Greenfield, IN) and Monovet 90 (**MV**; Huvepharma, Peachtree City, GA). The MV source recently came to market in 2019, while RUM has been on the market since 1975.

Feeding cattle steam-flaked corn (**SFC**) has been reported to improve ruminal starch digestion (Zinn, 1987), increase passage rate, and reduce CH_4_ production (Johnson et al., 1968; Zinn, 1987), resulting in improved efficiency of feed utilization (Zinn, 1987). If steam-flaking is unavailable, most commercial feedlots will feed dry-rolled corn (**DRC**) or high moisture corn. Also, in early monensin sodium research, it was tested in diets with less readily fermentable carbohydrates (DRC vs. SFC) feedlot diets. In order to properly assess both monensin sodium sources, both corn processing methods were included in this study. Our hypothesis was that there would be no differences between monensin sodium source, the negative control would have increased DMI, and acetate to propionate ratio (**A:P**). Further we predicted that CH_4_ levels would be similar between monensin sodium sources, and higher for the negative control. Furthermore, the cattle consuming the DRC diet would have increased DMI compared to the SFC diet. Therefore, the objective of this study was to evaluate the ruminal fermentation characteristics of ruminally cannulated beef steers consuming a SFC- or DRC-based diet containing either RUM or MV.

## MATERIALS AND METHODS

All experimental procedures involving live animals were approved (Protocol number 2020.01.001) by the Animal Care and Use Committee at West Texas A&M University and adhered to the regulations in the *Guide for the Care and Use of Agricultural Animals in Agricultural Research and Teaching* (Federation of Animal Science Societies, 2010).

### Animals

Six crossbred beef steers (657.7 kg ± 72.6) were used in a 6 × 6 Latin square design with a factorial arrangement of treatments at the Texas A&M AgriLife/USDA-ARS Research Feedlot near Bushland, TX. Steers were fitted with a rumen cannula (10.2 cm, Bar Diamond, Inc., Parma, ID). The steers were randomly assigned to 1 of 6 treatments in each period used in this experiment. Each period was 15 d with 14 d for diet adaptation and wash out and 1 d of rumen fluid collections.

Steers were housed in individual pens (5 m wide × 12 m long), with access to an individual water and feed bunk to prevent treatment contamination. The dietary treatments were finishing diets containing; 1) DRC with Rumensin 90 (**DRC-R**; 30 g/T; Elanco, Greenfield, IN), 2) DRC with Monovet 90 (**DRC-MV**; 30 g/T; Huvepharma, Peachtree City, GA), 3) SFC with Rumensin 90 (**SFC-R**; 30 g/T), 4) SFC with Monovet 90 (**SFC-MV**; 30 g/T), 5) DRC without monensin sodium (**DRC-C**), and 6) SFC without monensin sodium (**SFC-C**; Table 1). All diets included cottonseed meal, corn stalks, cane molasses, corn oil, and a supplement premix (Table 1). The supplement premix containing dried distiller’s grains (**DDG**), limestone, urea, vitamin/mineral supplement, treatment additives were mixed by hand into either the DRC or SFC basal diet prior to each feeding. Separate mixing tubs were used for each treatment to avoid contamination.

**Table 1.** Ingredient composition and calculated nutrient composition of treatment diets

	Dietary treatments^1^					
Item, % (DM basis)	SFC-C	SFC-R	SFC-MV	DRC-C	DRC-R	DRC-MV
Steam-flaked corn	70.0	70.0	70.0	-	-	-
Dry-rolled corn	-	-	-	70.5	70.5	70.5
Cottonseed meal	9.5	9.5	9.5	9.0	9.0	9.0
Corn stalks	10.0	10.0	10.0	10.0	10.0	10.0
Cane molasses	4.0	4.0	4.0	4.0	4.0	4.0
Corn oil	1.5	1.5	1.5	1.5	1.5	1.5
Treatment supplement^2^	5.0	5.0	5.0	5.0	5.0	5.0
Nutrient values^3^						
DM, %	76.95	76.33	76.95	76.40	77.06	77.40
CP, %	14.08	14.08	14.08	14.13	14.13	14.13
Ether extract, %	4.06	4.06	4.06	4.09	4.09	4.09
NDF, %	17.51	17.51	17.51	18.58	18.58	18.58
Ca, %	0.63	0.63	0.63	0.65	0.65	0.65
P, %	0.30	0.30	0.30	0.33	0.33	0.33
S, %	0.15	0.15	0.15	0.14	0.14	0.14
Na, %	0.37	0.37	0.37	0.37	0.37	0.37
NEm, Mcal/kg	2.12	2.12	2.12	2.03	2.03	2.03
NEg, Mcal/kg	1.40	1.40	1.40	1.31	1.31	1.31

^1^Dietary treatments consisted of a steam-flaked corn finishing diet with no monensin (SFC-C), with Rumensin 90 (SFC-R; Elanco, Greenfield, IN), or with Monovet 90 (SFC-MV; Huvepharma, Peachtree City, GA); or a dry-rolled corn finishing diet with no monensin (DRC-C), with Rumensin 90 (DRC-R; Elanco, Greenfield, IN), or with Monovet 90 (DRC-MV; Huvepharma, Peachtree City, GA).

^2^Treatment supplement consisted of dried distiller’s grains, limestone, urea, vitamins, minerals, and respective treatment additive. Supplement was formulated to meet or exceed the vitamin and mineral recommendations from NASEM (2016). Supplement provided 30 g/ton of monensin, either Rumensin 90 or Monovet 90 for the respective treatment. The CON treatment supplement contained no monensin.

^3^Values based off proximate analysis of individual ingredients.

### Feeding

Before trial initiation, steers were transitioned from a forage-based diet to the respective finishing treatment diet and fed ad libitum. Steers were fed twice daily at approximately 0700 h (deemed h 0) and 1100 h (deemed h 4). The premix was weighed to the nearest 0.1 kg for DDG, limestone, urea, vitamin/mineral supplement and the monensin supplement (RUM or MV) to the nearest 0.01 g. Diets were weighed to the nearest 0.1 kg daily.

### Diet Sample Collection and Analyses

Diet samples were collected directly from the bunk during each morning feeding of the adaptation period (d 1 to 14). Additionally, 3 individual diet samples were collected on d 15 (rumen fluid collection d). The d 1 to 14 diet samples were composited by treatment for each period. Half of the composited d 1 to 14 samples were dried at 55 °C, and half was stored at 4 °C for further analysis. One d 15 diet sample was dried at 55 °C and the other 2 samples were frozen at 4 °C for further analysis. Daily feed refusals were removed from the bunk, weighed, dried at 55 °C, and composited by treatment for each period. Feed was also collected and deemed a refusal if there were excess fines or whole corn stalks present, or when the feed was deemed soiled due to precipitation or animals defecating in the bunk. Daily DMI was quantified by subtracting refusals from the prior day’s feeding. Feed was removed and bunks were cleaned at the beginning of each period to prevent cross contamination between treatment diets.

### In Vivo Sample Collection

Rumen contents were collected through the fistula of each animal at 0, 3, 6, 12, and 24 h on d 15 of each period. Ruminal pH was measured (symphony H10p; VWR International, Radnor, PA) directly inside the rumen at the time of collection and contents were strained through 4 layers of cheese cloth, and four 50 mL tubes were stored at 4 °C for subsequent determination of total and molar proportion of volatile fatty acid (**VFA**) and ammonia concentration.

### In Vitro Digestion

Rumen fluid also was collected on d 15 of each period for in vitro analyses of dietary treatments. Rumen fluid (1 L) was collected 6 h post first feeding (~1300 h) from each of the 6 steers and strained through 4 layers of cheese cloth, placed directly into a pre-warmed, insulated container for transport to the laboratory. The treatment dietary substrates used were composited (d 1 to 7) during each period, dried for 48 h at 55 °C, and ground through a 2 mm screen (Thomas-Wiley Laboratory Mill Model 4; Thomas Scientific, Swedesboro, NJ). Ground samples were weighed (0.5 g) directly into a 250 mL glass bottle (Ankom Technology, Fairport, NY) in quadruplicate. Samples were inoculated with 150 mL of a 2:1 mixture of McDougal’s buffer and rumen fluid. Rumen fluid, McDougal’s buffer, and inoculum were measured for pH using an electronic pH meter (symphony B10p; VWR International, Radnor, PA) prior to incubation. Inoculant treatment fluid was used with its respective treatment substrate in vitro. After inoculation, the bottle was flushed with carbon dioxide, capped with a pressure module (Ankom Technology), a gas collection bag (FlexFoil PLUS Sample Bag; SKC Inc., Eighty Four, PA) was secured, and the bottle was placed into an incubator shaker (G25, New Brunswick Scientific Co. Inc., Edison, NJ). Samples were incubated for 48 h with continuous agitation in a shaker box set to 40 °C. The bottles were incubated for 48 h. Total gas production was measured using the Ankom RF Gas System (Ankom Technology) and recorded hourly. All gasses produced were composited into one gas collection bag over the 48 h time period for CH_4_ analysis at a later time.

### In Vivo Laboratory Analysis

Individual feed ingredients were sampled weekly for dry matter (**DM**). Feed ingredient DM was determined by drying samples at 55 °C for 48 h. Weekly ingredient samples were composited by month and sent to a commercial laboratory (Servi-tech Laboratories, Amarillo, TX) for nutrient analysis. Diet samples were analyzed for DM, ash, neutral detergent fiber (**NDF**), acid detergent fiber (**ADF**), crude protein (**CP**), fat, and starch. The NE_m_ and NE_g_ values were calculated utilizing tabular value from actual ingredient inclusion in the diet (Preston, 2016; Table 1).

Rumen fluid samples were analyzed VFA as described previously (Erwin, 1961; Ottenstein, 1971; Egert-McLean, 2020). Briefly, samples were thawed, centrifuged (5 min at 2,000 × *g*), and pipetted (1 mL) and into microcentrifuge tubes and combined with internal standard (100 μL 85 mM 2-ethylbutyrate) and deproteinizing agent (100 μL 50% meta-phosphoric acid). Tubes were mixed for approximately 5 s using a vortex and frozen (−4 °C) to allow for protein precipitation. Tubes were thawed, centrifuged at 39,000 × *g* for 20 min, and the supernatant was transferred to gas chromatography (**GC**) injection vials and capped. Gas chromatography with a flame ionization detector (Agilent HP6890 Plus GC with Agilent 7683 Series Injector and Auto Sampler; Agilent Technologies, Santa Clara, CA) and a Supelco 25326 Nukol fused silica capillary column (15 m × 0.53 mm × 0.5 μmol L^−1^ film thickness; Sigma/Supelco, Bellefonte, PA) were used to determine VFA concentrations in the rumen fluid samples. Analysis involved injection of 0.2 μL of each sample in duplicate at 110 °C with a 2:1 split, a 1 min hold, temperature increase at 5 °C min to 125 °C for 2 min, and the set point for inlet and injector at 260 °C. Molar percentage was calculated by dividing concentration of the VFA but the concentration of the total VFA and multiplying by 100. Samples were run with blanks and in duplicate. If the CV was greater than 5%, samples were rerun until CV was less than 5%.

Rumen fluid samples prepared as described above were analyzed for ammonia concentration, using a commercial kit (Sigma cat. No. K-3752, (800) 325–3010) employing glutamate dehydrogenase (Sigma cat. No. G-2626) and adapted for use on a Konelab 20XTi Analyzer (Thermo Electron Corporation, Waltham, MA). Samples were run with blanks and in duplicate. If the CV was greater than 5%, samples were rerun until CV was less than 5%.

### In Vitro Laboratory Analysis

Concentrations of CH_4_ were determined on a Varian 450 GC with a flame ionization detector (**FID**) (Varian, Inc., Palo Alto, CA) and a CombiPal auto sampler (CTC Analytics, Zwingen, Switzerland). A 2 mL subsample was auto injected, delivering 500 μL to the FID. The system was configured with a 0.5 m HayeSep N backflush column (Hayes Separations, Inc., Bandera, TX) followed by a 2 m Poropak QS analytical column. Calibration curves were developed using commercial blends of CH_4_ in air (Airgas Specialty Gases, Durham, NC).

### In Vivo and In Vitro Statistical Analysis

Data were analyzed as an incomplete-balanced Latin square design with a factorial arrangement of treatments using the PROC MIXED procedure in SAS (SAS Inst., Inc., Cary, NC). While the study was designed to be a complete Latin square, two steers had incomplete data due to multiple losses of the steer’s cannulas and rumen contents. One steer’s rumen fluid was not collected on period 4, 5, or 6 due to low dietary treatment intake (<6.8 kg DMI) due to loss of cannula multiple times. The other steer missed a 24-h data point due to loss of cannula and no rumen contents present. Data from both steers were removed completely.

Steer was the experimental unit. The model included fixed effects of corn grain processing, additive, and corn grain processing × additive. Period was considered a random effect. Ruminal pH, VFA, and CH_4_ concentration were analyzed as repeated measures in SAS. Ruminal pH data were analyzed using h as a repeated measure, and the time under a pH of 5.2 was determined using the MESS package of R (R Core Team, 2014), assuming a cubic spine interpolation and using 5.6 as the base pH. The LSMEANS statement with PDIFF option was used to separate treatment means. Effects were considered significant at *P*-value of ≤0.05, with tendencies declared at *P* > 0.051 and *P* ≤ 0.10.

## RESULTS AND DISCUSSION

### In Vivo

The average monensin intake for DRC-R treatment was 375.75 mg/steer-1 d-1, 378.5 mg/steer-1 d-1 for DRC-MV, 398.7 mg/steer-1 d-1 for SFC-R, and 391.5 mg/steer-1 d-1 for SFC-MV, respectively. There was no significant interaction for DMI (*P* = 0.49). Additive treatment (*P* = 0.81) nor grain processing (*P* = 0.58) influenced DMI in the present experiment (Table 2). It has been well documented that monensin sodium decreases feed intake while increasing ADG (Zinn, 1987; Stock et al., 1995; Duffield et al., 2012). Schelling et al. (1984) indicated that this increase in DMI could be due to an increase in ruminal turnover rate. This increase in ruminal turnover rate is caused by monensin sodium changing the rumen microbe population and density (Schelling et al., 1984). Further, Teixeira et al. (2020) also reported no difference (*P* = 0.37) in DMI for Nellore bulls consuming high concentrate diet with either no monensin sodium (7.35 kg) compared to RUM (7.13 kg) and another monensin sodium product (Shandong Qilo King-Phar Pharmaceutical Co. ltd., Jinan, China; 6.99 kg). However, Hales et al. (2017) reported an increase in DMI for steers fed monensin sodium when comparing a DRC finishing diet void of or containing 400 mg monensin/steer. Zinn (1987) reported that when comparing DRC and SFC-based finishing diets, cattle eating SFC ate significantly less compared to DRC. In the present study no difference was observed in DMI due to additive treatment (monensin sodium or no monensin sodium) or the type of grain fed (DRC vs. SFC). Steers used in this trial may have developed some resistance to monensin sodium from previous studies conducted when monensin sodium was present in the diet. Monensin sodium resistance has also been reported by Bergen and Bates (1984), Russell and Strobel (1989), and Russell and Houlihan (2003). Further, there may have not been enough replication to see a difference in DMI in the present study.

**Table 2.** The effect of type of monensin on dry matter intake, ruminal pH, and volatile fatty acid concentrations in cannulated beef steers

	Additive^1^				Grain^2^			*P-*Value		
Item	CON	Rumensin 90	Monovet 90	SEM	SFC	DRC	SEM	Additive	Grain	Additive × Grain
Steers, n	4	4	4	-	4	4	-	-	-	-
DMI, kg	11.94	11.71	11.64	0.341	11.87	11.65	0.282	0.81	0.58	0.49
Ruminal pH	5.90	5.88	5.96	0.068	5.98	5.85	0.065	0.33	≤0.01	0.62
Time under 5.6 pH, h	11.45	10.44	10.80	1.999	10.65	11.14	1.712	0.93	0.82	0.20
Time under 5.2 pH, h	1.52	3.58	0.19	0.875	3.53	0.00	0.69	0.03	≤0.01	0.03*
NH_3_, mg/dL	7.73	8.59	7.68	8.590	6.03	9.97	1.497	0.65	≤0.01	0.31
Total VFA, mM	118.60	116.52	111.74	6.529	110.39	120.85	5.555	0.73	0.16	0.79
VFA, mol/100 mol										
Acetate	51.16	51.94	50.50	-	50.42	51.98	-	0.34	0.07	0.36
Propionate	33.56	33.88	35.65	-	36.61	32.12	-	0.09	≤0.01	0.86
Butyrate	9.91^a^	9.19^b^	8.66^b^	-	7.93	10.58	-	≤0.01	≤0.01	0.49
Valerate	2.43	2.52	2.51	-	2.56	2.41	-	0.91	0.44	≤0.01^†^
Isobutyrate	0.75	0.63	0.74	-	0.68	0.73	-	0.31	0.50	0.73
Isovalerate	2.29	1.87	1.89	-	1.93	2.11	-	0.24	0.45	0.95
A:P^3^	1.59	1.67	1.44	-	1.46	1.67	-	0.06	0.01	0.71

^1^Additives were included in a basal finishing diet with either no additive (CON), Rumensin 90 (Elanco, Greenfield, IN), or Monovet 90 (Huvepharma, Peachtree City, GA).

^2^Diets had 2 different grain processing types, either steam-flaked corn (SFC) or dry-rolled corn (DRC).

^3^A:P = Acetate-to-propionate ratio.

^a–b^Treatment means without a common superscript differ (*P* ≤ 0.05).

**Figure 1.**
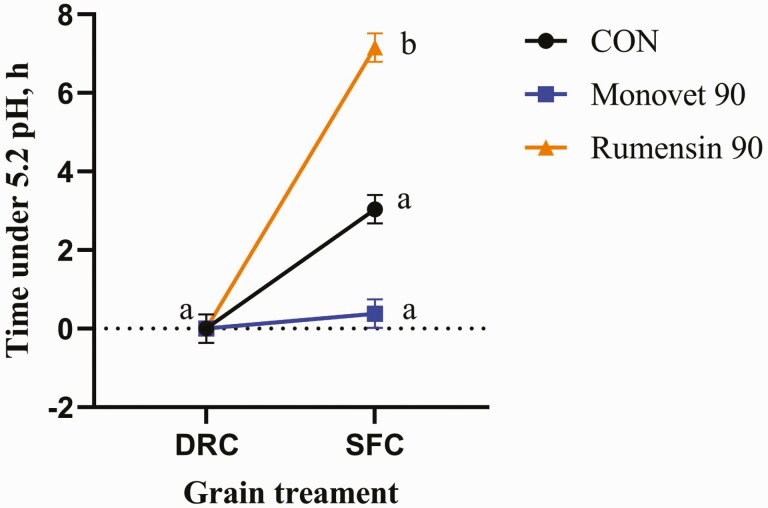
Time (h) under 5.2 pH showed a significant interaction for the additive by grain treatments (*P* ≤ 0.01). Grain treatments were dry-rolled corn (DRC) and steam-flaked corn (SFC) based finishing diets. The additive treatments were no monensin sodium (CON), Rumensin 90 (Elanco, Greenfield, IN), or Monovet 90 (Huvepharma, Peachtree City, GA). The interaction means were as follows; 0.0 for DRC by CON, 0.0 for DRC by MV, 0.0 for DRC by RUM, 3.0 for SFC by CON, 0.4 for SFC by MV, and 7.2 for SFC by RUM, respectively. ^a–b^Treatment means without a common superscript differ (*P* ≤ 0.05).

^*^Interaction means in Fig. 1.

**Figure 2.**
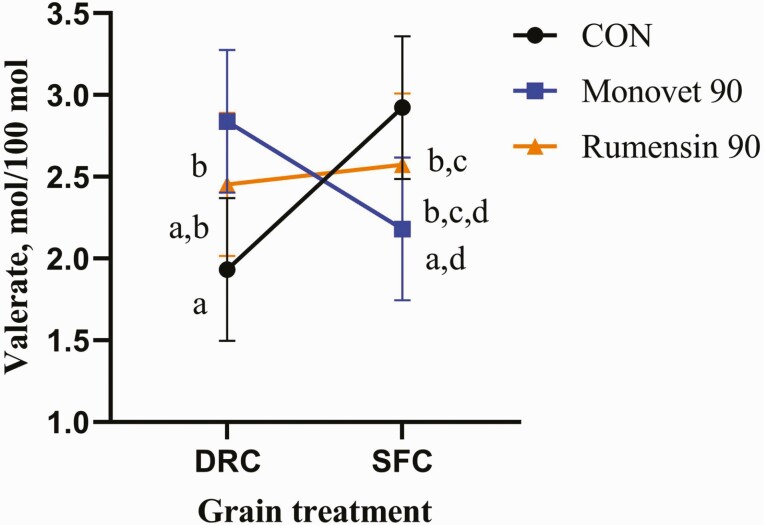
Valerate, mol/100 mol, had a significant interaction for the additive by grain treatments (*P* ≤ 0.01). Grain treatments were dry-rolled corn (DRC) and steam-flaked corn (SFC) based finishing diets. The additive treatments were no monensin sodium (CON), Rumensin 90 (Elanco, Greenfield, IN), or Monovet 90 (Huvepharma, Peachtree City, GA). The interaction means were as follows; 1.9 for DRC by CON, 2.8 for DRC by MV, 2.5 for DRC by RUM, 2.9 for SFC by CON, 2.2 for SFC by MV, and 2.6 for SFC by RUM, respectively. ^a–d^Treatment means without a common superscript differ (*P* ≤ 0.05).

^†^Interaction means in Fig. 2.

There was no significant interaction for ruminal pH (*P* = 0.62). There was a significant decrease in ruminal pH from the SFC grain treatment compared to the DRC treatment (*P* ≤ 0.01; Table 2). There was no significant difference in ruminal pH across the additive treatments (*P* = 0.33). The daily duration (h) that ruminal pH was below a 5.6 threshold did not differ across additive treatments (*P* = 0.93; Table 2) or grain treatments (*P* = 0.82). However, there was a significant interaction for duration of time ruminal pH was under a 5.2. Interaction means are presented in Fig. 1. The DRC treatment spent 0 h and SFC treatment spent between 3 and 7.2 h under the 5.2 pH. Jennings et al. (2020) reported that cattle consuming a SFC finishing diet spent between 0.81 and 3.23 h under a 5.4 pH threshold.

There was no significant interaction between additive and grain treatments for ruminal ammonia (**NH**_**3**_) concentration (*P* = 0.31). There were no significant differences between NH_3_ concentrations for the additive treatments (*P* = 0.65; Table 2). However, there was a significant increase in NH_3_ concentration for the DRC treatment compared to the SFC treatment (*P* ≤ 0.01; Table 2). May et al. (2009) reported greater NH_3_ concentrations for cattle fed DRC-based finishing diets (73.8% inclusion) compared to SFC-based finishing diets. Also, Cooper et al. (2002) reported comparable results that cattle fed a DRC diet had greater ruminal NH_3_ concentrations compared to the SFC diet at 12 h post feeding. This can be explained by greater ruminal starch fermentation, increased microbial growth, and assimilation of ruminal N (Cooper et al., 2002; May et al., 2009).

There was no significant interaction between the additive and grain treatments for total VFA produced (*P* = 0.79). There were no differences in the total amount of VFA (mM) produced for the additive treatments (*P* = 0.73; Table 2) or grain treatments (*P* = 0.16). Similarly, Johnson et al. (1968) reported no differences between SFC and DRC finishing diet’s total VFA concentrations (*P* > 0.05).

There were no significant interactions between additive and grain treatments for acetate (*P* = 0.36), propionate (*P* = 0.86), butyrate (*P* = 0.49), isobutyrate (*P* = 0.73), or isovalerate (*P* = 0.71). Furthermore, there were no differences for acetate (*P* = 0.34), valerate (*P* = 0.91), isobutyrate (*P* = 0.31), or isovalerate (*P* = 0.24) for the additive treatments. Propionate tended to be lower for the CON treatment (33.56 mol/100 mol; *P* = 0.09) compared to the two monensin sodium treatments (33.88 vs. 35.65 mol/100 mol, respectively; Table 2). Butyrate was least (*P* ≤ 0.01) for the MV treatment (8.66 mol/100 mol; Table 2), followed by the RUM treatment (9.19 mol/100 mol), and greatest for CON (9.91 mol/100 mol; *P* ≤ 0.01). The A:P tended to be less for MV compared to the CON and RUM treatments (*P* = 0.06).

There tended to be a decrease in acetate production (*P* = 0.07) in the cattle consuming SFC compared to DRC (Table 2). The DRC treatments had lesser propionate (*P* ≤ 0.01) compared to the SFC treatment. Butyrate levels were lower for the SFC treatment (*P* ≤ 0.01) than the DRC treatment. There was a significant interaction for valerate (*P* ≤ 0.01). Interaction means are presented in Fig. 2. There were no differences between isobutyrate (*P* = 0.50; 0.68 vs. 0.73 mol/100 mol, respectively) and isovalerate (*P* = 0.45; 1.93 vs. 2.11 mol/100 mol, respectively) for SFC and DRC. The A:P was lowest for the SFC treatment compared to the DRC treatment (*P* = 0.01). The data in this study coincides with Zinn (1987) and May et al. (2009) who reported a reduction in A:P for a SFC finishing diet compared to a DRC diet.

### In Vitro

**Figure 3.**
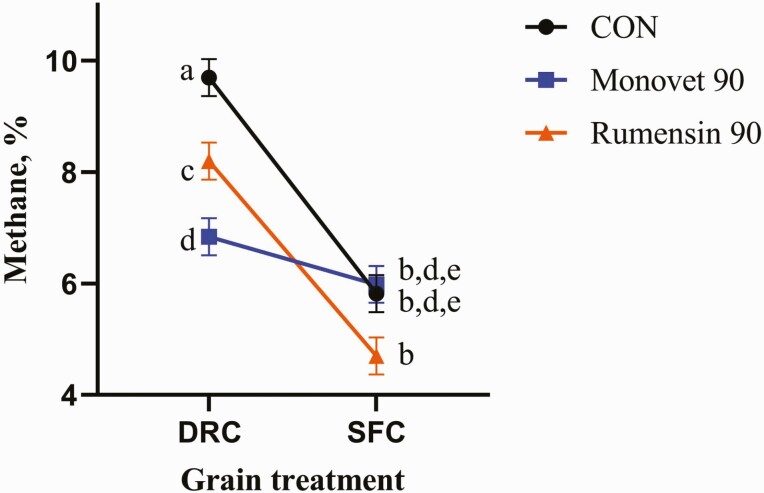
Methane, %, had a significant interaction for the additive by grain treatments (*P* ≤ 0.01). Grain treatments were dry-rolled corn (DRC) and steam-flaked corn (SFC) based finishing diets. The additive treatments were no monensin sodium (CON), Rumensin 90 (Elanco, Greenfield, IN), or Monovet 90 (Huvepharma, Peachtree City, GA). The interaction means were as follows; 9.70 for DRC by CON, 6.84 for DRC by MV, 8.20 for DRC by RUM, 5.82 for SFC by CON, 5.98 for SFC by MV, and 4.70 for SFC by RUM, respectively. ^a–e^Treatment means without a common superscript differ (*P* ≤ 0.05).

There was a significant interaction for total CH_4_ produced (*P* ≤ 0.01). There were also significant interactions between additive and grain treatments. The interaction means are presented in Fig. 3. Methane production was greatest (*P* ≤ 0.01) after 48 h incubation for the DRC-C treatment (9.70%) compared to other treatments, respectively (Fig. 3). Mechior et al. (2018) did not report a change in CH_4_ or CO_2_ for monensin compared to control heifers in vivo. However, Thornton and Owens (1981) reported that monensin sodium decreased CH_4_ production by 16% for two lower roughage level treatments (14 and 46.2% cottonseed hulls DM basis, respectively) and by 24% for the high roughage level treatment (65.7% cottonseed hulls DM basis) in vivo. These reported decreases in CH_4_ production coincided with an increase in ruminal propionate concentrations (Thornton and Owens, 1981). Due to the grain processing for SFC, starch is more readily available (Zinn, 1990; Zinn et al., 2002) which results in increased digestion and passage rate, thus leading to less methanogenic bacteria and overall less CH_4_ production (Johnson et al., 1968; Zinn, 1990; Zinn et al., 2002). Hales et al. (2012) reported that steers consuming DRC diets (73% inclusion DM basis) produced significantly more CH_4_ than SFC-based diets (73% inclusion DM basis; 74.31 vs. 58.77 L/steer CH_4_, respectively). Zinn et al. (1987) also reported a decrease in CH_4_ production for SFC compared to DRC in a finishing diet.

## IMPLICATIONS

Steer daily DMI did not differ for the additive or grain treatments. It is known that including monensin sodium in the diet most likely decreases DMI which should be considered when feeding monensin sodium commercially. Monensin sodium source did not affect ruminal pH, NH_3_, or total VFA production and both sources decreased CH_4_. Overall, there were minimal differences between monensin sources, and either could be used commercially with similar outcomes.

## References

[CIT0001] Bergen, W. G., and D. B. Bates. 1984. Ionophores: their effect on production efficiency and mode of action. J. Anim. Sci. 58:1465–1483. 10.2527/jas1984.5861465x 6378864

[CIT0002] Cooper, R. J., C. T. Milton, T. J. Klopfenstein, T. L. Scott, C. B. Wilson, and R. A. Mass. 2002. Effect of corn processing on starch digestion and bacterial crude protein flow in finishing cattle. J. Anim. Sci. 80:797–804. doi:10.2527/2002.803797x 11890417

[CIT0003] Dennis, S. M., T. G. Nagaraja, and E. E. Bartley. 1981. Effect of lasalocid or monensin on lactate-producing or using rumen bacteria. J. Anim. Sci. 52: 418–426. 10.2527/jas1981.522418x 7275867

[CIT0004] Duffield, T. F., J. K. Merrill, and R. N. Bagg. 2012. Meta-analysis of the effects of monensin in beef cattle on feed efficiency, body weight gain, and dry matter intake. J. Anim. Sci. 90:4583–4592. doi:10.2527/jas.2011-5018 22859759

[CIT0005] Egert-McLean, A. M., M. P. Sama, J. L. Klotz, N. B. Kristensen, and D. L. Harmon. 2020. Effects of a moderate transition from 70% to 90% concentrate diet on early alterations in feeding behavior, rumen environment, reticulorumen motility, and blood acid-base status in beef heifers. Can. J. Anim. Sci. 00:1–11. 10.1139/cjas-2019-0218

[CIT0006] Ellis, J. L., J. Dijkstra, A. Bannink, E. Kebreab, S. E. Hook, S. Archibeque, and J. France. 2012. Quantifying the effect of monensin dose on the rumen volatile fatty acid profile in high-grain-fed beef cattle. J. Anim. Sci. 90:2717–2726. doi:10.2527/jas.2011-3966 22896736

[CIT0007] Erwin, E. S., G. J. Marco, and E. M. Emery. 1961. Volatile fatty acid analyses of blood and rumen fluid by gas chromatography. J. Dairy. Sci. 44:1768–1770. DOI:10.3168/jds.S0022-0302(61)89956-6

[CIT0008] Federation of Animal Science Societies. 2010. Guide for the care and use of agricultural animal in research and teaching, 3rd ed. Federation of Animal Science societies, Campaign, IL. https://www.fass.org/images/science-policy/Ag_Guide_3rd_ed.pdf (Accessed 19 October, 2020).

[CIT0009] Hales, K. E., N. A. Cole, and J. C. MacDonald. 2012. Effects of corn processing method and dietary inclusion of wet distillers grains with solubles on energy metabolism, carbon-nitrogen balance, and methane emissions of cattle. J. Anim. Sci. 90;174–3185, 10.2527/jas.2011-4441 22585790

[CIT0010] Hales, K. E., J. E. Wells, E. D. Berry, N. Kalchayanand, J. L. Bono, and M. Kim. 2017. The effects of monensin in diets fed to finishing beef steers and heifers on growth performance and fecal shedding of *Escherichia coli* O157:H7. J. Anim. Sci. 95:3738–3744. doi:10.2527/jas.2017.1528 28805884

[CIT0011] Jennings, J. S., C. L. Lockard, L. O. Tedeschi, and T. E. Lawrence. 2020. Effects of corn stalk inclusion rate on rumination and ruminal pH in finishing beef steers. App. Anim. Sci. 36:377–388. 10.15232/aas.2019-01947

[CIT0012] Johnson, D. E., J. K. Matsushima, and K. L. Knox. 1968. Utilization of flaked vs. cracked corn by steers with observations on starch modification. J. Anim. Sci. 27:1431–1437. 10.2527/jas1968.2751431x

[CIT0014] May, M. L., M. J. Quinn, C. D. Reinhardt, L. Murray, M. L. Gibson, K. K. Karges, and J. S. Drouillard. 2009. Effects of dry-rolled or steam-flaked corn finishing diets with or without twenty-five percent dried distillers grains on ruminal fermentation and apparent total tract digestion. J. Anim. Sci. 87: 3630–3638. 10.2527/jas.2008-0857 19648506

[CIT0500] McGuffey, R. K., L. F. Richardson, and J. I. D. Wilkinson. 2001. Ionophores for dairy cattle: current status and future outlook. J. Dairy Sci. 84:194–203. doi:10.3168/jds.S0022-0302(01)70218-411210033

[CIT0015] Melchior, E. A., K. E. Hales, A. K. Lindholm-Perry, H. C. Freetly, J. E. Wells, C. N. Hemphill, T. A. Wickersham, J. E. Sawyer, and P. R. Myer. 2018. The effects of feeding monensin on rumen microbial communities and methanogenesis in bred heifers fed in a drylot. Livestock Sci. 212: 131–136. 10.1016/j.livsci.2018.03.019.c

[CIT0016] National Academics of Sciences, Engineering, and Medicine (NASEM). 2016. Nutrient requirements of beef cattle, eighth revised edition. Washington, DC: The Nationals Academics Press.

[CIT0017] Ottenstein, D. M., and D. A. Bartley. 1971. Separation of free acids C2-C5 in dilute aqueous solution column technology. J. Chromatographic Sci. 9:673–681. 63. 10.1093/chromsci/9.11.673

[CIT0018] Preston, R. L . 2016. 2016 feed composition table BEEF Magazine. https://www.beefmagazine.com/datasheet/2016-beef-feed-compostition-table-pdf-download. (Accessed May 2, 2021).

[CIT0019] R Core Team. 2014. R: A language and environment for statistical computing. R Found. Stat. Com. Vienna, Austria.

[CIT0020] Russell, J. B., and A. J. Houlihan. 2003. Ionophore resistance of ruminal bacteria and its potential impact on human health. FEMS Microbiol Rev. 27:65–74. 10.1016/S0168-6445(03)00019-6 12697342

[CIT0021] Russell, J. B., and H. J. Strobel. 1989. Effect of ionophores on ruminal fermentation. Appl. Environ. Microbiol. 55:1–6. doi:10.1128/AEM.55.1.1-6.1989 2650616 PMC184044

[CIT0022] Schelling. G. T . 1984. Monensin mode of action in the rumen. J. Anim. Sci. 58:1518–1527. 10.2527/jas1984.5861518x 6378867

[CIT0023] Stock, R. A., S. B. Laudert, W. W. Stroup, E. M. Larson, J. C. Parrott, and R. A. Britton. 1995. Effect of monensin and monensin and tylosin combination on feed intake variation of feedlot steers. J. Anim. Sci. 73:39–44. doi:10.2527/1995.73139x 7601752

[CIT0024] Teixeira, D. A. A., B. I. Cappellozza, J. R. Fernandes, K. S. Nascimento, L. E. L. M. Bonfim, C. N. Lopes, J. A. C. Ehrhardt, J. R. Peres, S. A. Harris, J. M. C. Simas, et al. 2020. Effects of monensin source on in vitro rumen fermentation characteristics and performance of *Bos indicus* beef bulls offered a high-concentrate diet. Trans. Anim. Sci. 4:84–94. 10.1093/tas/txz158 PMC6994034 32704969

[CIT0025] Thornton, J. H., and F. N. Owens. 1981. Monensin supplementation and in vivo methane production by steers. J. Anim. Sci. 52: 628–634, 10.2527/jas1981.523628x 6267003

[CIT0026] Wood, K. M., A. C. Pinto, D. D. Millen, R. Kanafany Guzman, and G. B. Penner. 2016. The effect of monensin concentration on dry matter intake, ruminal fermentation, short-chain fatty acid absorption, total tract digestibility, and total gastrointestinal barrier function in beef heifers. J. Anim. Sci. 94:2471–2478. doi:10.2527/jas.2016-0356 27285923

[CIT0027] Zinn, R. A . 1987. Influence of lasalocid and monensin plus tylosin on comparative feeding value of steam-flaked versus dry-rolled corn in diets for feedlot cattle. J. Anim. Sci. 65:256–266. doi:10.2527/jas1987.651256x 3610873

[CIT0028] Zinn, R. A . 1990. Influence of steaming time on site of digestion of flaked corn in steers. J. Anim. Sci. 68: 776–781. 10.2527/1990.683776x 2318739

[CIT0029] Zinn, R. A., F. N. Owens, and R. A. Ware. 2002. Flaking corn: processing mechanics, quality standards, and impacts on energy availability and performance of feedlot cattle. J. Anim. Sci. 80:1145–1156. doi:10.2527/2002.8051145x 12019600

